# High-Strength Nanotwinned Copper Combined with Silicon/Silicon Nitride/Graphite Anode for High-Performance Lithium-Ion Battery

**DOI:** 10.3390/ma19122496

**Published:** 2026-06-10

**Authors:** Fu-Chian Chen, Rahmandhika Firdauzha Hary Hernandha, Dinh-Phuc Tran, Jeng-Kuei Chang, Chih Chen

**Affiliations:** 1 Department of Materials Science and Engineering, National Yang Ming Chiao Tung University, Hsinchu 300093, Taiwan; fuchianchen.mse@gmail.com (F.-C.C.); hary.hernandha@gmail.com (R.F.H.H.); 2 MATCHA ROOM^®^, Jl. Monginsidi, Pacul Permai A-19, Bojonegoro 62114, East Java, Indonesia; 3 Graduate Institute of Manufacturing Technology, National Taipei University of Technology, Taipei 106344, Taiwan; dptran@ntut.edu.tw; 4 Department of Chemical Engineering and R&D Center for Membrane Technology, Chung Yuan Christian University, 200 Chung Pei Road, Taoyuan 32023, Taiwan

**Keywords:** artificial graphite, copper foil, high-rate retention, lithium-ion battery, mechanical properties, silicon nitride

## Abstract

Graphite is widely used as an anode in lithium-ion batteries (LIBs); however, its limited capacity restricts the energy density enhancement. Si-based anodes offer much higher capacity, but their significant volume changes during repeated lithiation and delithiation generate mechanical stress and can damage the electrode/current-collector interface. Herein, high-strength nanotwinned Cu (NT-Cu) was fabricated and employed as current collectors for carbon-coated Si/β-Si_3_N_4_-based anodes. The electroplated 5 μm-thick NT-Cu foils exhibited tensile strength exceeding 760 MPa. The role of the Cu current collector was investigated by comparing NT-Cu foils with different mechanical properties and commercial Cu foils. The results show that electrochemical performance was not governed by UTS alone; instead, a balanced combination of tensile strength, ductility, and surface morphology was important for improving cycling stability and rate capability. To further improve cycling retention, artificial graphite was incorporated into the Si/β-Si_3_N_4_ composite. Using a 5 μm electroplated NT-Cu foil and a Si/β-Si_3_N_4_/artificial graphite composite anode, the pouch cell retained 81.51% of its capacity and delivered 267.3 mAh g^−1^ after 206 cycles. These results demonstrate the potential of NT-Cu for improving the stability of Si-containing LIB anodes.

## 1. Introduction

The unprecedented growth of electric vehicles and electronic markets has driven an increasing demand for high-energy-density LIBs [1,2,3,4,5,6]. Additionally, the demand for medical treatment wearable devices is rising due to the population aging alongside life-enhancing technologies. To meet these needs, electronic devices are required to be compact and multifunctional, which brings an inevitable challenge—the short lifetime of batteries [1,2,3,4,5,6,7,8,9].

LIBs are the most essential energy storage components due to their high energy density, long cycle life, and low toxicity [1,2,9,10]. LIBs commonly use a carbon anode, which is mainly composed of graphite [11]. Despite some advancements in LIBs over the decades, carbon anode LIBs are increasingly unable to meet the needs of high energy density and lifetime [12]. However, the capacity of graphite is limited to 372 mAh g^−1^, which restricts further improvement in energy density. Replacing the graphite anode with other high-capacity anodes such as Si, Sn, Sb, Ge, Al, Mg, Bi, and some metal compounds (oxides, sulfides, phosphides) is one of the solutions. Among these candidates, Si is considered one of the most promising anode materials because of its high theoretical capacity, natural abundance, and potential for high-energy-density LIBs [13,14,15].

Si-based anodes are expected to show high energy density, eco-friendliness, and low cost for LIB mass production. Although Si-based anodes offer significantly higher capacity, their application is limited by significant volume changes during lithiation, which remains one of the major challenges for Si-containing LIB anodes [13,14,15]. The repeated expansion/contraction induces substantial mechanical stresses, leading to particle fracture, loss of electrical contact, and rapid capacity fading.

In addition to active-material design, the current collector has recently been recognized as an important component affecting the rate capability, cycling stability, and interfacial reliability of LIB electrodes [16]. This role becomes particularly important for Si-containing anodes, where repeated volume variation can continuously transfer stress to the collector interface. Insufficient mechanical compatibility at this interface can cause delamination, increased interfacial resistance, and accelerated performance degradation. Despite this important role, the mechanical contribution of the Cu current collector has received comparatively limited attention [9,17,18,19,20]. Tetsu Kiyobayashi et al. [21] demonstrated that using a high-strength Cu-Ni-Cu clad foil with 920 MPa ultimate tensile strength (UTS) as a current collector outperformed regular Cu foil. Using a high-strength Cu foil can avoid severe wrinkles, which can prevent the reduced electrical contact between the active material and the current collector. Therefore, improving the mechanical robustness of the Cu current collector is a rational strategy for maintaining electrode integrity in Si-based anodes. In addition to mechanical strength, the surface morphology of the Cu current collector can also affect the electrode/current-collector interface. For example, roughened Cu current collectors have been reported to improve adhesion between Si-based electrodes and Cu foils, leading to enhanced cycling behavior [22]. Therefore, both mechanical robustness and surface morphology should be considered when designing Cu current collectors for Si-containing anodes.

Nanotwinned Cu (NT-Cu) has high tensile strength, high thermal stability, low resistivity, and high electromigration resistance [23,24,25,26]. It can be deposited by two different processes: magnetron sputtering and electroplating. Although sputtering can deposit Cu with extremely high twin density and highly preferred orientation, its low deposit rate raises the cost for mass production. A recent research on nanotwinned Cu foils for Li batteries compares Li-metal batteries made of [111] oriented nanotwinned Cu foils to those made with polycrystalline Cu foils. It is found that the rough surface of the [111]-oriented nanotwinned Cu foil favors a homogeneous Li nucleation, attributing to excellent coulombic efficiency (CE) and high capacity retention of the Li-metal batteries [23,27,28,29,30,31,32,33,34,35,36,37].

For the Si/Si_3_N_4_-based composite anode for LIBs, there are only a few studies on crystalline Si_3_N_4_ as a matrix. Zhang et al. conducted research using crystalline Si_3_N_4,_ which reached initial capacities of ~800 and ~1000 mAh/g with very low initial coulombic efficiency (ICE). Even though they applied a low current rate (0.08 A/g), their optimum composite (Si/a-Si_3_N_4_p) could only stand for 50 cycles with ~47% capacity retention [38]. Kim et al. deposited a Si/Si_3_N_4_ composite on the carbon nanofibers (CNFs) using an electrospinning process and polyacrylonitrile as a precursor for graphitic carbon forming. They could achieve an ICE of ~61.3% and the first reversible capacity of ~610 mAh/g [39]. In early 2020, a Si@α-Si_3_N_4_@C composite made by two-step gas–solid reaction showed a high initial reversible capacity. However, after 200 cycles, the capacity retention was revealed as only 86.5% (in ~0.89 A/g). It might be attributed to the fact that the architecture design of Si_3_N_4_ was applied as a shell, and its ratio was only ~33%. Thus, the Si_3_N_4_ shell could not sufficiently prevent the volume expansion of the Si core [40]. In the present Si/β-Si_3_N_4_ composite system, β-Si_3_N_4_ is introduced mainly as a structurally stable matrix and stress-buffering phase rather than as a primary conductivity enhancer. The β-Si_3_N_4_ phase can dilute the effective Si content and help alleviate the mechanical instability associated with Si volume variation during cycling. The electronic conduction pathway is mainly supported by the carbon coating and conductive carbon additive. Therefore, the role of β-Si_3_N_4_ in this work is to improve the structural stability and stress tolerance of the Si-containing composite anode.

In contrast to previous studies that mainly focused on Si-based active materials, carbon coatings, binders, or electrolyte modification, this work emphasizes the mechanical role of the Cu current collector in Si-containing composite anodes. Electroplated NT-Cu foils were used not only as conductive substrates but also as mechanically reinforced current collectors for carbon-coated Si/β-Si_3_N_4_-based electrodes. The novelty of this study lies in correlating the tensile properties and surface morphology of 5 μm-thick NT-Cu foils with the rate capability and cycling behavior of Si/β-Si_3_N_4_-based anodes. By comparing NT-Cu foils with different mechanical states and commercial Cu foils, this work suggests that the electrochemical response of Si/β-Si_3_N_4_-based electrodes is not governed by UTS alone but is associated with the combined effects of tensile strength, ductility, surface morphology, and preservation of electrode/current-collector contact.

## 2. Materials and Methods

### 2.1. Rotary Electroplating Nt-Cu Foils for Current Collectors

**Figure 1:**
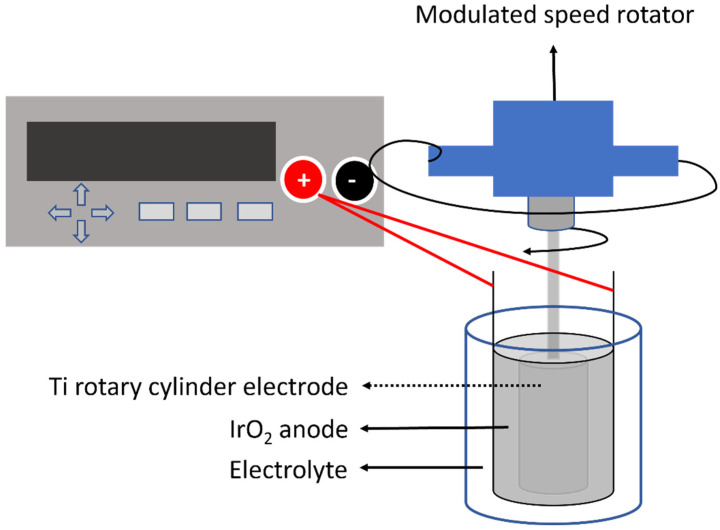
Schematic of the rotary electroplating system.

Rotary electroplating was conducted to deposit NT-Cu foils. Figure 1 shows the illustration of the experimental setup. The cylinder Ti cathode was locked on a modulated speed rotator and rotated at 800 rpm, which was surrounded by a Ti-coated iridium dioxide hollow cylinder anode. The electrolyte contained 0.8 M Cu sulfate (CuSO_4_), 40 ppm chloride ion, 100 g of sulfuric acid, and DP101 additive (Chemleader Inc., Hsinchu, Taiwan).

Three types of NT-Cu were used in this study: NT-Cu A, NT-Cu B, and NT-Cu C. Two commercially available Cu foils were used as benchmark current collectors and are denoted as Commercial A and Commercial B. Their manufacturer names are not disclosed due to commercial confidentiality. The main differences between these two commercial foils are their thickness and ductility: Commercial A is a 10 μm-thick Cu foil with an elongation of 3.2%, whereas Commercial B is a thinner 6 μm-thick Cu foil with an elongation of 1.9%. Their UTS values are similar, 485 MPa for Commercial A and 492 MPa for Commercial B, as summarized in Table 1. Three types of NT-Cu foils were prepared by adjusting the electroplating current density while keeping the other bath conditions identical. NT-Cu A, NT-Cu B, and NT-Cu C were deposited at current densities of 30, 20, and 11 ASD, respectively. The different current densities resulted in NT-Cu foils with different tensile properties and surface morphologies, which were further compared as current collectors for Si/β-Si_3_N_4_-based anodes.

**Figure 2:**
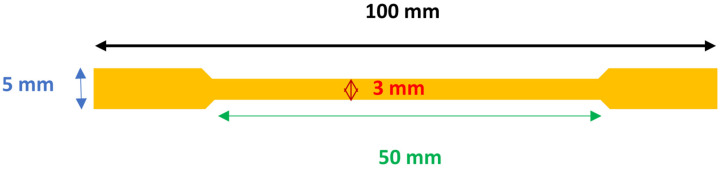
Outline dimension drawing of a dog bone-shaped tensile test sample.

The formation of nanotwinned Cu during electroplating is strongly affected by deposition kinetics and crystallographic growth behavior. During high-current-density deposition, the increased cathodic overpotential leads to rapid nucleation and suppresses lateral grain growth. The presence of organic additives such as DP101 modifies the adsorption behavior at the cathode surface, leading to more uniform current distribution. Therefore, the combined effects of kinetic control and additive-assisted growth contribute to the formation of nanotwinned Cu columnar microstructures in this study. Five dog bone samples (Figure 2) were cut from the 5 cm × 12 cm electrodeposited Cu foils. The mechanical properties were characterized by a tensile test machine (AGS-X, Shimadzu, Kyoto, Japan) with a strain rate of 4.17 × 10^−3^ at room temperature.

### 2.2. Fabrication of Cr2032 Coin Cells Using Carbon-Coated Si/β-Si_3_N_4_ Composite Active Materials and Nt-Cu Foils

**Figure 3:**
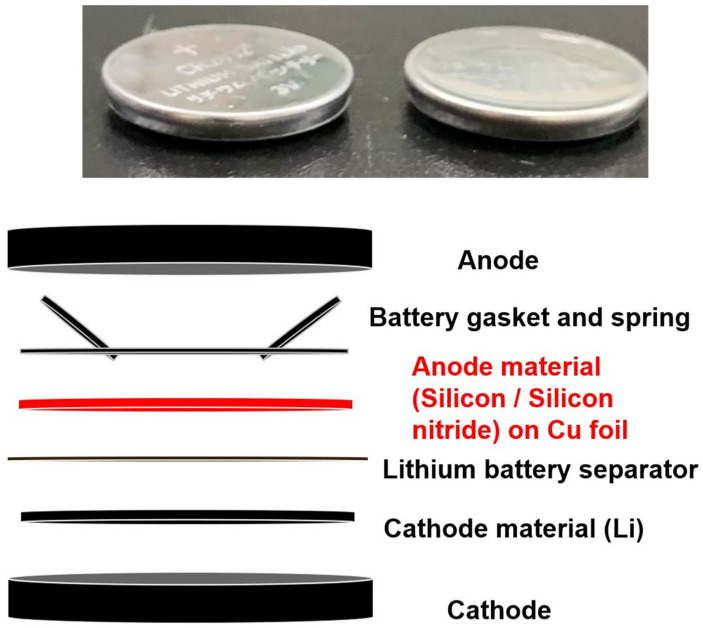
Schematic configuration and photograph of the CR2032 coin-type half-cell, in which Li foil was used as the cathode.

Commercial micro-sized Si powder (average D_50_: 1.8 μm, purity > 99.9%) and β-Si_3_N_4_ powder (average D_50_: ~16 μm, purity > 99.9%) were provided by Super Energy Material Inc., Taoyuan, Taiwan. Different compositions of Si powder and β-Si_3_N_4_ have been examined in this study. Based on the reported synthesis procedure [38], after ball milling and drying processes, a wet-chemical mixing was adopted as a carbon coating protocol. Anhydrous ethanol and glucose were used as the solvent and carbon precursor, respectively. Finally, the carbonization was conducted at 850 °C under Ar for 5 h. The final particle size of carbon-coated Si/β-Si_3_N_4_ composites in various Si/β-Si_3_N_4_ ratios is ~300–500 nm. The carbon-coated Si/β-Si_3_N_4_, Super P, and sodium polyacrylate binder (7:2:1 ratio) were mixed in deionized water, and they were coated on the NT-Cu foil using a stainless-steel blade. Then the specimen was dried at 100 °C for 6 h under vacuum. After drying, it was cut into the size of a CR2032 coin cell battery. The active material mass loading was ~2 mg cm^−2^. The Li foil cathode and glass-fiber membrane separator are shown in Figure 3. An electrolyte consisting of 1 M LiPF_6_, ethylene carbonate (EC)/diethyl carbonate (DEC) solvent (1:1 by volume), and 5 wt.% fluoroethylene carbonate (FEC) additive was used. The coin cells were fabricated in an Ar-filled glove box. Previous studies using the same ball-milling and carbon-coating process have confirmed the coexistence of Si/β-Si_3_N_4_ phases through Raman spectra, XRD, TEM, SEM, and XPS [41].

### 2.3. Preparation of Pouch Cell Using Carbon-Coated Si/β-Si_3_N_4_ Composite Active Materials and Nt-Cu Foils

For the full-cell fabrication, a selected AG-containing carbon-coated Si/β-Si_3_N_4_ composite anode and an NT-Cu foil current collector were adopted. The full cell (3 × 58 × 102 mm) was fabricated by Gold Carbon Co., Ltd., Taiwan, China. The NMC-622 was adopted as the cathode material. Using 3.0–4.3 V as operating voltage (OCV: 3.76 V), the pouch cell was run at a current rate of 1.0 C, with a mode of CC-CV charge and CC discharge. The anode-to-cathode capacity (N/P) ratio is 1.2. The cell was conditioned within 3.6–4.3 V at 0.1 C for 3 cycles.

## 3. Results and Discussion

### 3.1. Mechanical Properties of Nt-Cu Foils

**Figure 4:**
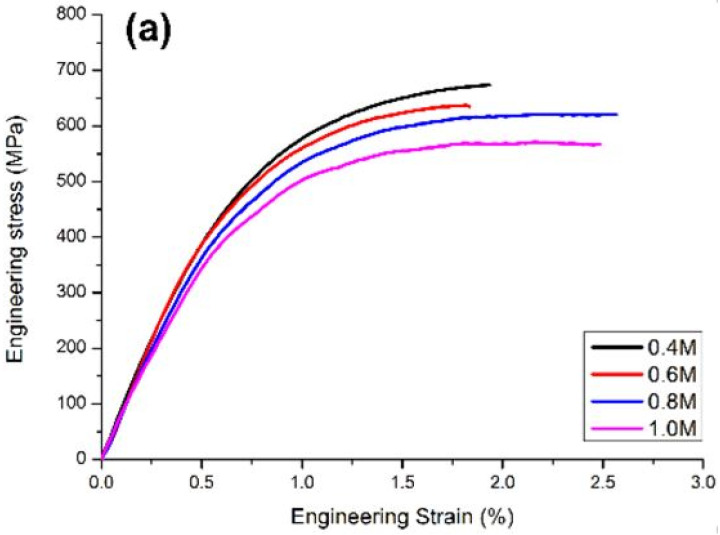

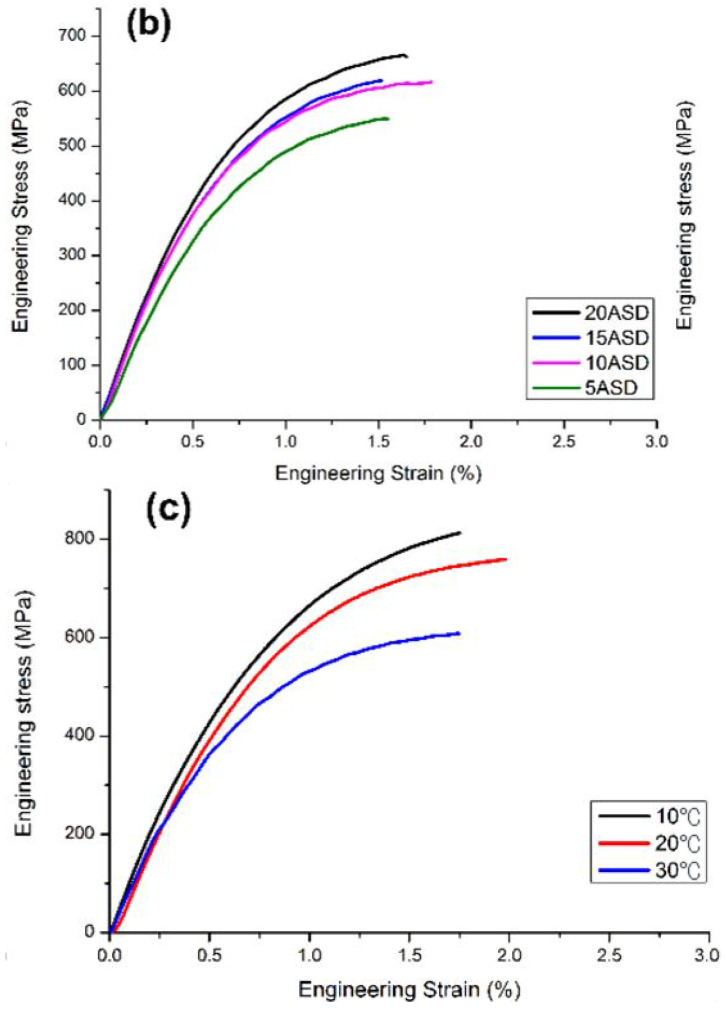
The trend of tensile strength related to different electroplating parameters: (**a**) Cu ion concentration, (**b**) electroplating current density, (**c**) electrolyte temperature.

**Figure 5:**
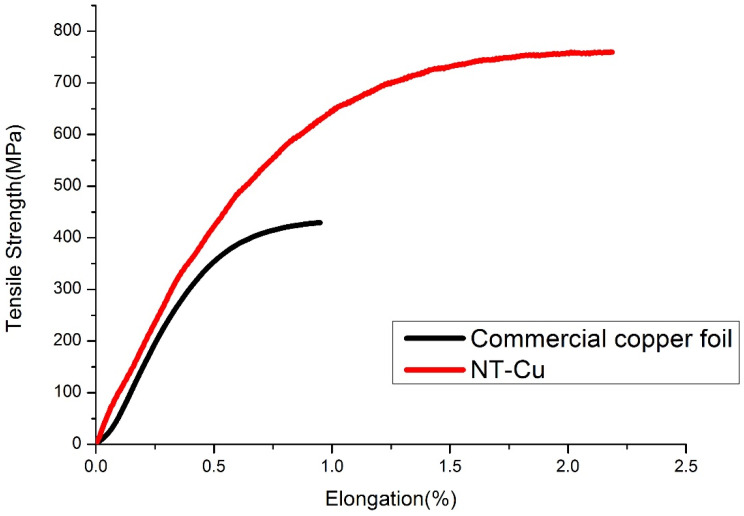
Comparing the most strengthening NT-Cu foils with commercial copper foils.

Figure 4 shows the effects of Cu ion concentration, current density, and electrolyte temperature on the tensile strength of 5 μm thick NT-Cu foils. As the Cu ion concentration decreases, the ultimate tensile strength increases, as illustrated in Figure 4a. In addition, the tensile strength increases as the electroplating current density increases, as depicted in Figure 4b. Furthermore, when the electrolyte temperature is lowered, the tensile strength increases, as presented in Figure 4c. However, it is noteworthy to state that reasonable elongation is needed for the NT-Cu foil to increase its toughness to withstand the charge/discharge-induced stresses [42]. The observed increase in UTS under reduced Cu^2+^ concentration, elevated current density, and decreased electrolyte temperature can be attributed to microstructural refinement and enhanced twin boundary density induced by kinetic control during electroplating. In addition, lowering the electrolyte temperature reduces atomic surface diffusion and grain-boundary migration, effectively suppressing grain growth and stabilizing twin structures during deposition. The combined effects of these parameters result in a refined microstructure with increased boundary density, which contributes to strength enhancement through boundary-strengthening mechanisms. It is worth noting that excessive refinement or overly high internal stress may deteriorate ductility and interfacial stability in battery applications. Therefore, an optimized balance between strength and ductility is critical for achieving improved electrochemical performance, as demonstrated by the superior behavior of NT-Cu B in subsequent battery tests. We adjusted the electroplating parameters to obtain the optimal mechanical strength of the Cu foil. Figure 5 shows the NT-Cu foil fabricated at 30 ASD, 0.8 M Cu sulfate, 40 ppm chloride ion, 100 g sulfuric acid, and 40 mL DP101 additives dissolved in 1L deionized water at 20 °C. The ultimate tensile strength is 760 MPa with 2.2% elongation. For comparison, we also fabricated NT-Cu of different mechanical properties for high-performance Li-ion batteries.

### 3.2. Cycling Test and Performance of Si/β-Si_3_N_4_/nt-Cu Foil Cr2032 Coin Cell

To determine the ideal composition of C-Si/β-Si_3_N_4_, two types of commercial Cu foils with different thicknesses were used for the preliminary tests. The mechanical properties of each Cu foil are shown in Table 1.

According to the results shown in Table 2, the higher the proportion of Si in the active material, the higher the capacity is before 100 cycles, but lower cycle retention is observed. To balance both capacity and cycle retention, it led us to choose C-Si/50%β-Si_3_N_4_ as standard composite active materials for further experimentation. The charge–discharge properties were evaluated using a NEWARE CT-4000 battery tester (Neware Technology Limited, Shenzhen, Guangdong, China) at 25 °C, and the cycling/rate conditions used for Table 2 are specified in the table caption and the corresponding text. β-Si_3_N_4_/Si composites with various β-Si_3_N_4_-to-Si ratios were fabricated by high-energy ball milling. β-Si_3_N_4_ and Si powders at various weight ratios (β-Si_3_N_4_/Si = 25/75, 50/50, 75/25) were dispersed in anhydrous ethanol and transferred to a zirconia vessel for planetary ball milling (a powder/zirconia ball weight ratio of 1/10 was used). Carbon layers were deposited onto different powder samples through a wet-chemical coating approach. Glucose served as the carbon source, and subsequent carbonization was carried out at 850 °C for 5 h in an Ar atmosphere. To verify the reproducibility and reliability of the electrochemical performance, charge–discharge tests for each electrode were conducted in at least three independent trials. The performance deviation was typically within ≈5%, and the reported values represent the median of three independent measurements.

The data in Table 2 reveal an obvious trade-off between capacity and cycle stability with varying Si content. As the Si fraction increases, the reversible capacity significantly increases (95.0 → 1602.1 mAh g^−1^), while the capacity retention decreases (≈100% → 61.1%), reflecting the intensified volume expansion associated with higher Si content.

C-Si/50%β-Si_3_N_4_ was selected as the optimized composition for subsequent current collector comparisons, as it provides a balanced compromise between energy density and structural stability. The trend in Table 2 also clarifies the function of β-Si_3_N_4_ in the composite anode. Increasing the Si fraction enhances reversible capacity but decreases capacity retention, indicating that excessive Si content intensifies volume-expansion-induced instability. In contrast, introducing β-Si_3_N_4_ improves the structural stability of the composite by acting as a relatively stable matrix and stress-buffering component. Therefore, C-Si/50%β-Si_3_N_4_ was selected because it provides a practical balance between capacity contribution from Si and cycling stability supported by β-Si_3_N_4_.

**Figure 6:**
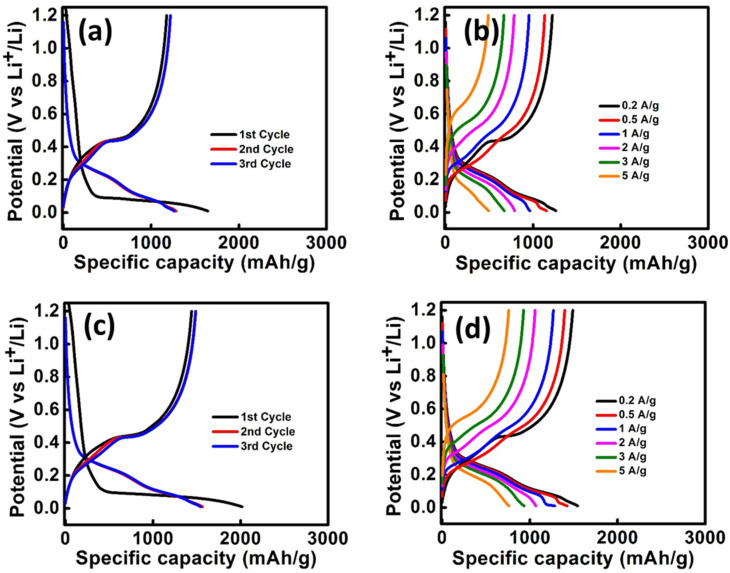
The charge–discharge curve of C-Si/50%β-Si_3_N_4_ with (**a**,**b**) Commercial A and (**c**,**d**) NT-Cu A.

**Figure 7:**
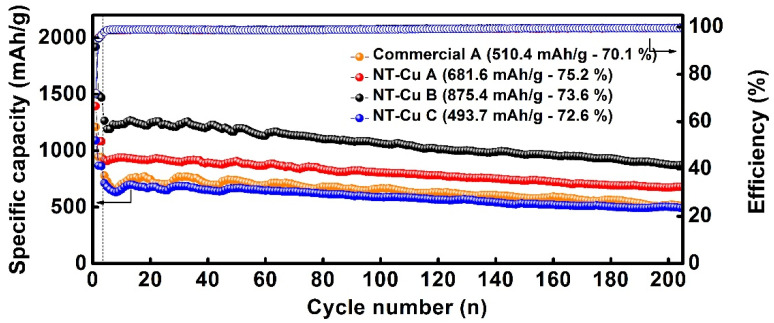
Cycle performance of C-Si/50%β-Si_3_N_4_ on NT-Cu and commercial copper foils at 1 A g^−1^ for 200 cycles.

Table 3 shows the capacity of coin cells made of three types of NT-Cu foils and two commercial Cu foils with different thicknesses at different current rates. The charge–discharge curve of commercial A and NT-Cu A is shown in Figure 6. As summarized in Table 3, NT-Cu foils deliver higher reversible capacities than commercial Cu foils at all tested current rates. For example, NT-Cu A shows 1015.2 mAh g^−1^ at 0.2 A g^−1^ and 517.6 mAh g^−1^ at 5 A g^−1^, compared with 751.9 and 264.0 mAh g^−1^ for Commercial A, respectively. The cycle retention of NT-Cu foil is also slightly better than that of the commercial Cu foil (Figure 7). The commercial A sample had a capacity of 510.4 mAh/g, with 70.1% retention after 200 cycles. The NT-Cu B sample performed the best capacity of 875.4 mAh/g, with 73.6% retention after 200 cycles. The capacity is 71.5% better than that of Commercial A, with a 3.5% increase in the retention after 200 cycles. By correlating the mechanical properties of the NT-Cu with the electrochemical results, it can be observed that optimal performance is achieved not at the highest tensile strength, but at an intermediate mechanical state. While NT-Cu A exhibits the highest UTS, NT-Cu B demonstrates superior rate capability and cycling stability, suggesting that balanced strength and ductility are critical for maintaining interfacial integrity under repeated mechanical stress. Therefore, in addition to UTS, elongation, strain-accommodation capability, and surface morphology should be considered as key Cu-foil factors affecting the electrochemical response of Si-containing electrodes.

**Figure 8:**
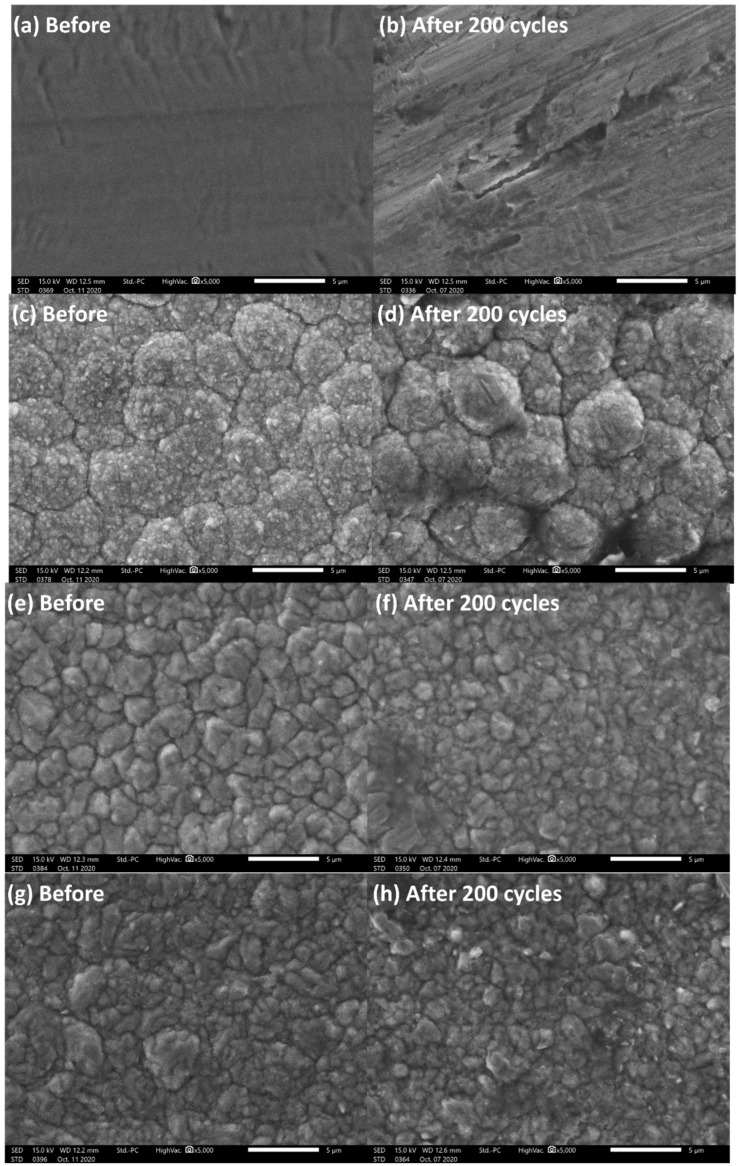
SEM images for the surface of Cu. (**a**) Commercial A, (**c**) NT-Cu A, (**e**) NT-Cu B, (**g**) NT-Cu C before cycle test. (**b**) Commercial A, (**d**) NT-Cu A, (**f**) NT-Cu B, (**h**) NT-Cu C after 200 cycles.

This indicates that excessive strengthening may reduce strain accommodation at the electrode–collector interface, whereas insufficient strength cannot effectively resist stress transfer from the expanding Si-based composite. Therefore, an optimized mechanical compatibility between the current collector and electrode plays a decisive role in electrochemical performance. To confirm the potential of high-tensile-strength NT-Cu foil for LIBs, we also conducted the same test with commercial Cu foil fabricated by international manufacturers—Commercial A and Commercial B in Table 4. Both the reversible capacity and high-rate retention indicate that the NT-Cu foils outperformed the commercial Cu foils. Commercial B was included in the rate-capability comparison, as shown in Table 3, to provide an additional commercial Cu foil benchmark. For the long-term cycling comparison in Figure 7, Commercial A was selected as the representative commercial Cu foil because it was the primary commercial reference foil used throughout this study and exhibited higher ductility than Commercial B. Therefore, the cycling test was focused on comparing the optimized NT-Cu foil with a representative commercial Cu current collector under the same electrode configuration. Figure 8 shows the SEM image of the Cu surface. Compared with NT-Cu A, NT-Cu B, and NT-Cu C, Commercial A exhibited more obvious surface cracking and damage after cycling, which is consistent with its poorer high-rate retention and cycling performance. After 200 cycles, the commercial Cu foil shows obvious surface cracking and structural damage, whereas NT-Cu foils maintain comparatively intact surface morphology. This structural integrity is consistent with the improved cycling stability and rate performance of NT-Cu-based electrodes, supporting the proposed mechanical stabilization effect of NT-Cu current collectors.

The electrochemical results obtained from C-Si/50%β-Si_3_N_4_ electrodes on different NT-Cu foils indicate that balanced foil characteristics, including UTS, elongation, and surface morphology, are important for this Si/β-Si_3_N_4_-based LIB system. Although high tensile strength is beneficial for mechanical support, the battery results show that UTS alone cannot fully determine the electrochemical performance of C-Si/β-Si_3_N_4_ electrodes.

Before we move to the final step to optimize and develop the LIB system in this study, we also attempted to analyze the surface morphology of NT-Cu foils. Three different NT-Cu foils, NT-Cu A, NT-Cu B, and NT-Cu C, which exhibited UTS values of 760, 560, and 535 MPa, respectively, were made into coin cells and tested. According to the ultimate tensile strength, we predicted that the NT-Cu A coin cell test should perform best; NT-Cu B and NT-Cu C coin cell tests should have similar behavior. The capacity performance for different currents is shown in Table 3. Surprisingly, NT-Cu B showed the best performance in both reversible capacity and high-rate retention among the three types of NT-Cu. NT-Cu B also showed an obvious difference compared with NT-Cu C. Although NT-Cu A exhibits the highest UTS, the electrochemical results indicate that NT-Cu B delivers the best overall cycling stability and rate capability. This result suggests that the improved performance is not solely determined by maximum strength, but rather by an optimized balance between strength and ductility.

NT-Cu A, with excessively high strength, may exhibit reduced strain-accommodation capability at the electrode–collector interface during repeated lithiation–delithiation cycles. In contrast, NT-Cu C, with relatively lower strength, may not provide sufficient mechanical support against stress transfer induced by the volume expansion of silicon. NT-Cu B achieves a balanced mechanical state, offering adequate structural reinforcement while maintaining interfacial compatibility.

Therefore, the superior performance of NT-Cu B originates from optimized mechanical compatibility rather than extreme strengthening alone. The following microstructural observations further support this interpretation.

**Figure 9:**
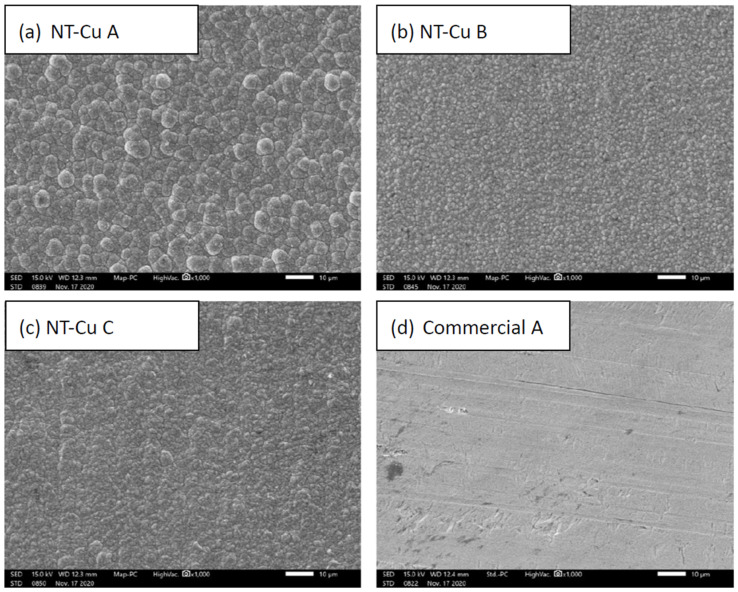
Surface morphology of Cu foils: (**a**) NT-Cu A, (**b**) NT-Cu B, (**c**) NT-Cu C, and (**d**) Commercial A.

**Figure 10:**
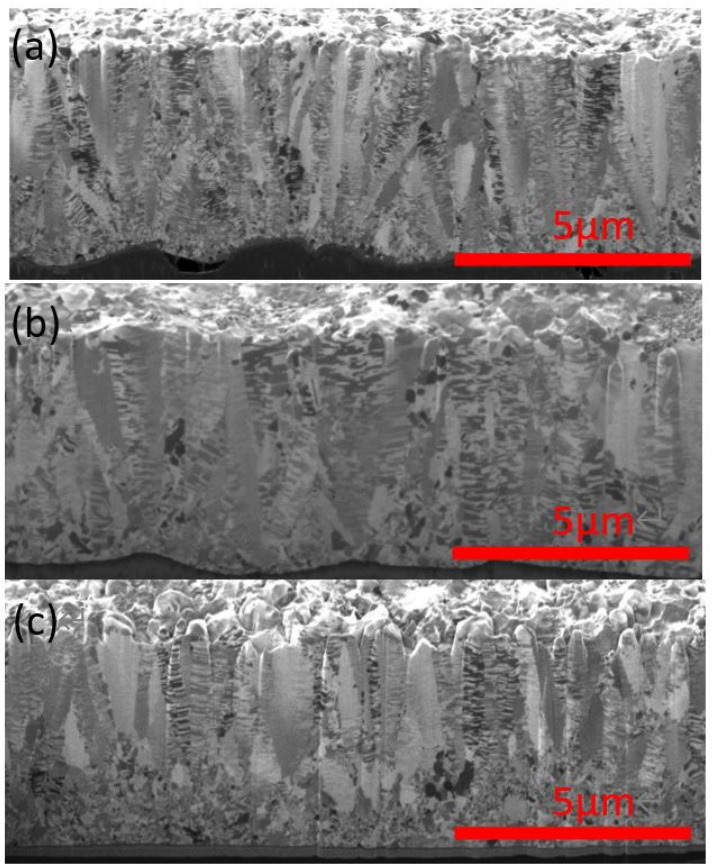
Cross-sectional ion image which shows the microstructure of (**a**) NT-Cu A, (**b**) NT-Cu B, (**c**) NT-Cu C.

**Figure 11:**
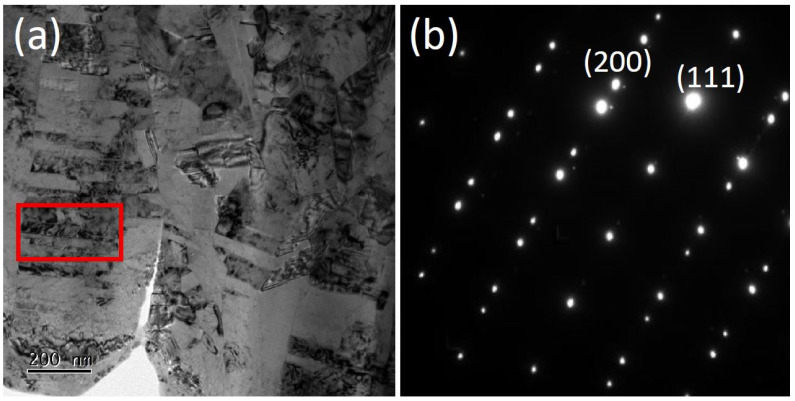
TEM image (**a**) of as-fabricated NT-Cu and corresponding selected area diffraction (SAD) patterns (**b**).

To further understand the structural origin of this optimized mechanical compatibility, the surface and cross-sectional morphologies were examined. The plan-view SEM images and the FIB cross-section ion images show obvious differences in surface morphology and microstructures in Figure 9 and Figure 10. In Figure 9a–c, it has been revealed that the NT-Cu A foil consisted of big grains, the NT-Cu B foil has small grains, and the NT-Cu C foil has large grains mixed with some small grains. And for the Commercial A foil, it is clearly shown in Figure 9d that the cold-rolled process tends to form a relatively flat surface. These observations indicate that the surface morphology of the Cu current collector is another factor influencing the electrochemical performance of C-Si/50%β-Si_3_N_4_ electrodes. This interpretation is consistent with previous reports showing that Cu current-collector morphology can affect adhesion with Si-based electrodes and consequently influence cycling behavior [22]. Therefore, the superior performance of NT-Cu B is attributed to the combined effects of tensile properties, strain-accommodation capability, and favorable surface morphology for electrode/current-collector contact rather than UTS alone. To further confirm the nanotwinned structure of the electroplated Cu foils, TEM and selected area diffraction (SAD) analyses were conducted, as shown in Figure 11. The TEM image reveals nanoscale lamellar features within the Cu grains, which are characteristic of nanotwinned structures. In addition, the SAD pattern can be indexed to face-centered cubic (FCC) Cu, with representative diffraction spots corresponding to the Cu (111) and (200) planes. The presence of multiple diffraction spots with slight orientation differences suggests the existence of twin-related domains within the selected area. These observations are consistent with previous reports on electrodeposited nanotwinned Cu foils fabricated under similar processing conditions.

### 3.3. Mechanical Stabilization Mechanism of Nt-Cu Current Collectors

Silicon-based anodes undergo severe volume expansion (~300%) during lithiation, which induces particle fracture, interfacial delamination, and repeated formation of unstable SEI layers. While most strategies focus on modifying active materials or binders, the mechanical contribution of the current collector is often underestimated.

In this study, the NT-Cu current collector enhances the mechanical robustness of the electrode–collector interface. The improved strength combined with maintained ductility allows the collector to better accommodate stress transfer during repeated lithiation–delithiation cycles. This reduces localized strain concentration and helps preserve electrical contact between the composite coating and the current collector.

The improved cycling stability and higher capacity retention observed for NT-Cu B suggest that the NT-Cu current collector helps preserve the mechanical integrity of the electrode/current-collector interface during cycling. This interpretation is supported by the rate-capability results, cycling retention, and post-cycling SEM observations. Since impedance measurements before and after cycling were not available in the present study, the discussion is limited to the mechanically supported evidence, and no direct conclusion is made regarding charge-transfer resistance or SEI chemistry. Further impedance analysis will be useful in future work to quantitatively evaluate interfacial resistance evolution during cycling.

### 3.4. Improving Cycle Retention by Incorporating Artificial Graphite into Si/β-Si_3_N_4_ Composites

**Figure 12:**
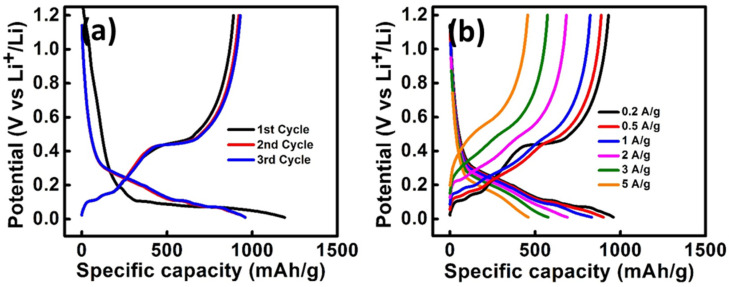
The charge–discharge curve of NT-Cu B (**a**) with C-Si/50%β-Si_3_N_4_/AG (**b**).

**Figure 13:**
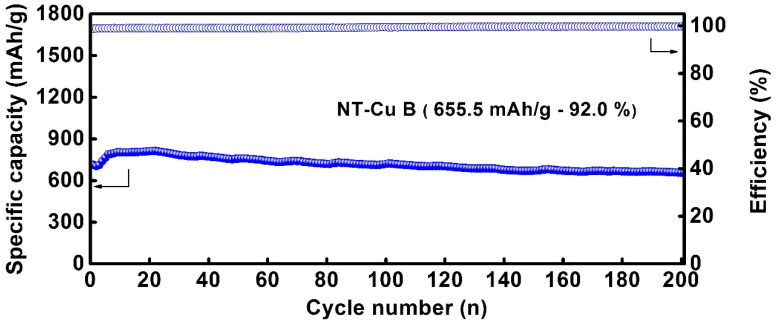
Cycle performance of C-Si/50%β-Si_3_N_4_/AG on NT-Cu B.

**Figure 14:**
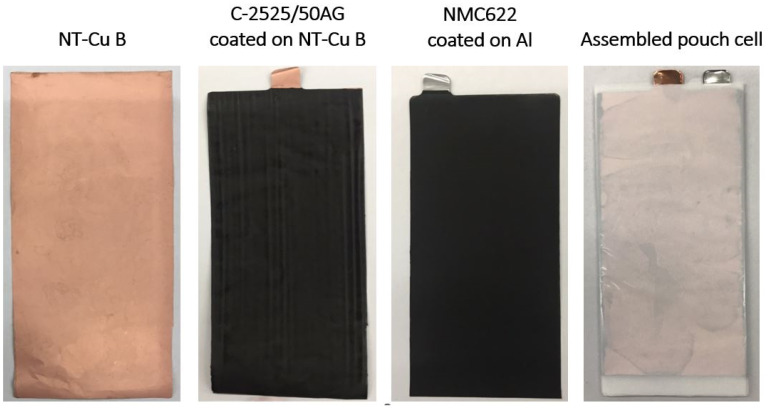
Pouch cell assembling schematic.

**Figure 15:**
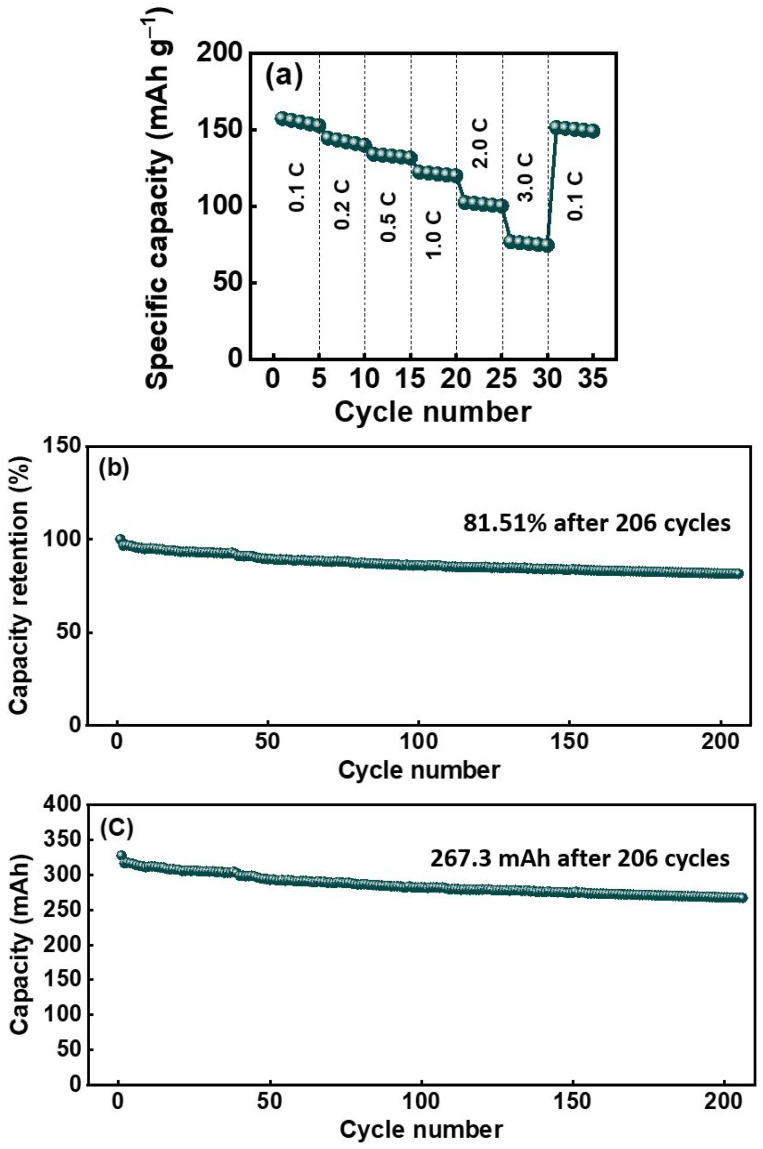
(**a**) The capacity at different current rates. (**b**,**c**) The cycle retention and capacity after 206 cycles.

C-Si/50%β-Si_3_N_4_ exhibited a high reversible capacity but showed only 71–75% capacity retention during cycling at 1 A g^−1^. To improve cycling stability, artificial graphite (AG) was incorporated into the C-Si/50%β-Si_3_N_4_ composite to form an AG-containing composite anode. Figure 12 and Figure 13 show the charge–discharge curves and cycling performance of the Si/50%β-Si_3_N_4_/AG electrode on NT-Cu B. With the incorporation of AG, the electrode achieved approximately 92% capacity retention after 200 cycles at 1 A g^−1^. This improvement indicates that AG can buffer the electrochemical and mechanical instability of the Si-containing composite by reducing the effective Si fraction and improving the overall cycling stability. However, the AG-containing composition used in this work should be regarded as a selected composition for demonstrating the feasibility of the NT-Cu/Si/β-Si_3_N_4_/AG design. A systematic optimization of the Si/β-Si_3_N_4_/AG ratio will be required in future work to further balance reversible capacity, retention, and electrode-level energy density. Based on these results, a pouch cell was fabricated using an NT-Cu foil current collector and a C-Si/50%β-Si_3_N_4_/AG composite anode, as illustrated in Figure 14. Figure 15a shows that the high-rate retention exceeded 60%. Figure 15b,c shows that the pouch cell maintained 81.51% capacity retention after 206 cycles, retaining a capacity of 267.3 mAh g^−1^. Although the retained capacity of 267.3 mAh g^−1^ is lower than the values commonly reported for Si-rich anodes in half-cell configurations, this value was obtained from a practical pouch-type full cell using an NMC-622 cathode, an N/P ratio of 1.2, and a Si/β-Si_3_N_4_/AG composite anode cycled at 1.0 C. In full-cell and pouch-cell configurations, the measured capacity is strongly affected by electrode balancing, cathode limitation, irreversible lithium consumption, and the dilution effect caused by introducing graphite to improve cycling stability. Therefore, the pouch-cell result should be interpreted as a demonstration of scalable cell feasibility and cycling stability rather than as an attempt to maximize the gravimetric capacity of the Si-based active material. Compared with previous reports on Si-containing full cells and pouch cells, the present cell delivers a moderate capacity but maintains 81.51% capacity retention after 206 cycles, indicating that the NT-Cu current collector and Si/β-Si_3_N_4_/AG composite design provide a stable full-cell configuration. However, the 206-cycle pouch-cell test should be regarded as an initial feasibility demonstration rather than a complete evaluation of commercial viability. Longer-term cycling tests under practical operating conditions will be required to further assess durability, degradation behavior, and commercial applicability.

## 4. Conclusions

We successfully electroplated 5 μm-thick NT-Cu foils with a UTS exceeding 760 MPa and an elongation of 2.2%. These NT-Cu foils were used as current collectors for Si/β-Si_3_N_4_-based LIB anodes. Compared with commercial Cu foils, the NT-Cu current collectors showed improved rate capability and cycling performance, suggesting their potential as mechanically reinforced current collectors for Si-containing anodes. However, the electrochemical performance was not determined by UTS alone. The results indicate that elongation, surface morphology, and electrode/current-collector contact stability also play important roles. Additionally, the combination of C-Si/50%β-Si_3_N_4_/AG composite as active material coated onto the high-strength NT-Cu foil resulted in a pouch cell exhibiting over 60% high-rate retention, and the cycle retention over 200 cycles still maintained 81.51% and a capacity of 267.3 mAh/g. These results highlight a current-collector engineering strategy for Si-containing anodes, showing that the mechanical state and surface morphology of NT-Cu foils can influence electrochemical performance beyond their conventional role as conductive substrates. Nevertheless, longer-term pouch-cell cycling and further optimization of the Si/β-Si_3_N_4_/AG composition are still required to evaluate practical durability and commercial viability.

## Figures and Tables

**Table 1:** Mechanical properties and thicknesses of NT-Cu foils and commercially available Cu foils used in this study.

	UTS (MPa)	Elongation (%)	Thickness (μm)
NT-Cu A	760	2.2	5
NT-Cu B	560	2.6	5
NT-Cu C	535	1.8	5
Commercial A	485	3.2	10
Commercial B	492	1.9	6

**Table 2:** The different composition of Si/β-Si_3_N_4_ and the related capacity and cycling retention performance.

Composition	Capacity After 100 Cycles (mAh/g)	Capacity Retention (%)
C-β-Si_3_N_4_	95.0	~100
C-Si/75%β-Si_3_N_4_	419.3	93.7
C-Si/50%β-Si_3_N_4_	716.0	76.9
C-Si/25β-Si_3_N_4_	1156.4	66.6
C-Si	1602.1	61.1

**Table 3:** Reversible capacities at different current rates for three types of NT-Cu and two different commercial Cu. The Coulombic efficiency of all results was greater than 99.9%.

Current Rate (A/g)	NT-Cu A (mAh/g)	NT-Cu B (mAh/g)	NT-Cu C (mAh/g)	Commercial A (mAh/g)	Commercial B (mAh/g)
0.2	1015.2	1488.8	859.5	751.9	823.2
0.5	957.6	1396.9	793.7	677.4	732.9
1	882.5	1267.6	703.2	574.9	612.4
2	741.8	1058.8	583.5	440.9	491.0
3	640.6	927.3	498.9	357.3	411.6
5	517.6	757.3	390.7	264.0	302.9
HR Ret (5/0.2)	50.9%	50.9%	45.4%	35.1%	36.7%

**Table 4:** Reversible capacities at different current rates for the NT-Cu B with C-Si/50%β-Si_3_N_4_/AG. The Coulombic efficiency of all results was greater than 99.9%.

Current Rate (A/g)	NT-Cu B (mAh/g)
0.2	929.8
0.5	887.4
1	822.3
2	682.6
3	571.8
5	455.8
HR Ret (5/0.2)	49%

## Data Availability

The original contributions presented in the study are included in the article; further inquiries can be directed to the corresponding authors.
